# Methods to Develop an Electronic Medical Record Phenotype Algorithm to Compare the Risk of Coronary Artery Disease across 3 Chronic Disease Cohorts

**DOI:** 10.1371/journal.pone.0136651

**Published:** 2015-08-24

**Authors:** Katherine P. Liao, Ashwin N. Ananthakrishnan, Vishesh Kumar, Zongqi Xia, Andrew Cagan, Vivian S. Gainer, Sergey Goryachev, Pei Chen, Guergana K. Savova, Denis Agniel, Susanne Churchill, Jaeyoung Lee, Shawn N. Murphy, Robert M. Plenge, Peter Szolovits, Isaac Kohane, Stanley Y. Shaw, Elizabeth W. Karlson, Tianxi Cai

**Affiliations:** 1 Division of Rheumatology, Brigham and Women’s Hospital, Boston, Massachusetts, 02115, United States of America; 2 Harvard Medical School, Boston, Massachusetts, 02115, United States of America; 3 Division of Gastroenterology, Massachusetts General Hospital, Boston, Massachusetts, 02114, United States of America; 4 Center for Systems Biology, Massachusetts General Hospital, Boston, Massachusetts, 02114, United States of America; 5 Department of Neurology, Brigham and Women’s Hospital, Boston, Massachusetts, 02115, United States of America; 6 Research Computing, Partners Healthcare, Charlestown, Massachusetts, 02125, United States of America; 7 Children’s Hospital Informatics Program, Boston Children’s Hospital, Boston, Massachusetts, 02115, United States of America; 8 Department of Biostatistics, Harvard School of Public Health, Boston, Massachusetts, 02115, United States of America; 9 Partners Healthcare Information Systems, Boston, Massachusetts, 02115, United States of America; 10 Tufts University School of Medicine, Boston, Massachusetts, 02111, United States of America; 11 Department of Neurology, Massachusetts General Hospital, Boston, Massachusetts, 02114, United States of America; 12 Division of Genetics, Brigham and Women’s Hospital, Boston, Massachusetts, 02115, United States of America; 13 Computer Science and Artificial Intelligence Laboratory (CSAIL), Massachusetts Institute of Technology, Cambridge, Massachusetts, 02139, United States of America; Laikon Hospital, GREECE

## Abstract

**Background:**

Typically, algorithms to classify phenotypes using electronic medical record (EMR) data were developed to perform well in a specific patient population. There is increasing interest in analyses which can allow study of a specific outcome across different diseases. Such a study in the EMR would require an algorithm that can be applied across different patient populations. Our objectives were: (1) to develop an algorithm that would enable the study of coronary artery disease (CAD) across diverse patient populations; (2) to study the impact of adding narrative data extracted using natural language processing (NLP) in the algorithm. Additionally, we demonstrate how to implement CAD algorithm to compare risk across 3 chronic diseases in a preliminary study.

**Methods and Results:**

We studied 3 established EMR based patient cohorts: diabetes mellitus (DM, n = 65,099), inflammatory bowel disease (IBD, n = 10,974), and rheumatoid arthritis (RA, n = 4,453) from two large academic centers. We developed a CAD algorithm using NLP in addition to structured data (e.g. ICD9 codes) in the RA cohort and validated it in the DM and IBD cohorts. The CAD algorithm using NLP in addition to structured data achieved specificity >95% with a positive predictive value (PPV) 90% in the training (RA) and validation sets (IBD and DM). The addition of NLP data improved the sensitivity for all cohorts, classifying an additional 17% of CAD subjects in IBD and 10% in DM while maintaining PPV of 90%. The algorithm classified 16,488 DM (26.1%), 457 IBD (4.2%), and 245 RA (5.0%) with CAD. In a cross-sectional analysis, CAD risk was 63% lower in RA and 68% lower in IBD compared to DM (p<0.0001) after adjusting for traditional cardiovascular risk factors.

**Conclusions:**

We developed and validated a CAD algorithm that performed well across diverse patient populations. The addition of NLP into the CAD algorithm improved the sensitivity of the algorithm, particularly in cohorts where the prevalence of CAD was low. Preliminary data suggest that CAD risk was significantly lower in RA and IBD compared to DM.

## Introduction

Electronic medical records (EMR) data are emerging as an important resource for large scale clinical and translational studies. A major challenge for studies using EMR data is assigning accurate phenotypes to millions of patients in a high throughput manner. The advancement of natural language processing (NLP) as well as the development of robust phenotyping methods [1, 2] now enables researchers to efficiently assemble cohorts of patients with specific diseases. Once the cohorts are assembled, attention is mainly focused on genetic and clinical association studies within the phenotype of interest [3, 4]. However, the data in the EMR can also support studies that compare and examine outcomes across disease cohorts. Thus there is now also a need for EMR phenotype algorithms that can perform well across populations where the patient characteristics may vary.

Few studies have addressed methods to classify an outcome across diverse disease cohorts. An algorithm that allows study of one phenotype across disease cohorts requires different specifications from existing phenotype algorithms. The main difference is that the algorithm must perform well when the prevalence for the phenotype of interest varies across cohorts. As an example, coronary artery disease (CAD) is highly prevalent in DM (21.9%)[5] compared to rheumatoid arthritis (RA) where the prevalence ranges from 5–10%[6]. A CAD algorithm developed in DM would likely perform poorly in RA where the prevalence of CAD is lower. Enriching the types of clinical data used in a phenotype algorithm may be one method to improve the portability of the algorithm across cohorts. Based on previous studies [7–9], we observed that including clinical data extracted using NLP significantly improved the performance of algorithms in different populations, particularly where the prevalence of the phenotype of interest was low.

The primary objective of this study was to develop a robust and accurate EMR CAD outcomes algorithm that can perform well across 3 established disease cohorts, DM, inflammatory bowel disease (IBD) and RA. Additionally, we tested whether algorithms incorporating NLP perform better than those developed using structured data alone. We hypothesize that NLP will be instrumental in improving the sensitivity of a CAD algorithm in RA and IBD where the prevalence is expected to be low compared to DM. We also demonstrated how to implement the algorithm by comparing the risk of CAD across DM, IBD and RA in a preliminary study. The results of the study may also inform current efforts to determine whether patients with inflammatory disease should be considered to have as much risk for CAD as patients with diabetes [10, 11]

## Methods

### Study design and setting

We studied data from 3 patient cohorts, DM, IBD and RA classified from the Partners EMR (containing over 4 million unique patients) with data from 1994–2010. Briefly, the cohorts were created using previously published EMR phenotype algorithms, incorporating a combination of structured data (e.g. ICD9 codes, electronic prescriptions, laboratory values) and clinical data extracted using NLP to mine the narrative text. The RA phenotype algorithm has a PPV of 94% and contains 4,453 subjects [8, 12]. Two algorithms were developed for IBD, one for Crohn’s disease (CD) and a second for ulcerative colitis (UC) with a total of 10,974 subjects. The PPV of the algorithms was 97% for both CD and UC, confirmed in an independent validation cohort [7]. Analogous methods were applied to develop the DM phenotype algorithm, containing 65,099 subjects validated with a PPV of 96% (Kumar, et al. in preparation). The goal of the IBD, RA and DM phenotype algorithms was to achieve high PPV (e.g. to identify subjects with definite disease), thus subjects with possible disease were excluded from the cohorts.

### Variables

#### Structured data

To create a list of potential structured data variables for a CAD phenotype algorithm, we obtained the ICD9 and CPT codes from the American Heart Association Get with The Guidelines—Coronary Artery Disease (AHA GTWTG-CAD)[13] (S1 Appendix).

#### Narrative data extracted using NLP

Three board certified cardiologists (RM, PN, RS in Acknowledgements) created a CAD list of terms (customized dictionary) using descriptions corresponding to ICD9 codes from the AHA GTWTG-CAD (S1 Appendix). In addition, they provided terms they used to describe a patient with CAD in their clinical notes. These terms were mapped to concepts, e.g. coronary artery bypass graft (CABG). The concepts were extracted from narrative text notes using the Health Information Text Extraction (HITex) system. HITex is an open source NLP tool which processes text notes and determines whether a concept of interest was mentioned in the note^18^. The output for this present study was whether a concept was mentioned (1 = yes, 0 = no).

### CAD algorithm

#### Outcome definition

CAD was defined as having a diagnosis of CAD in the medical record with supporting evidence of disease through documentation of CABG, percutaneous coronary intervention (PCI) with stent or balloon angioplasty, positive stress test, or EKG changes consistent ischemia. Subjects with a diagnosis of CAD without supporting documentation or subjects with no mention of CAD were considered not to have the disease.

#### Training and development- RA cohort

**Fig 1:**
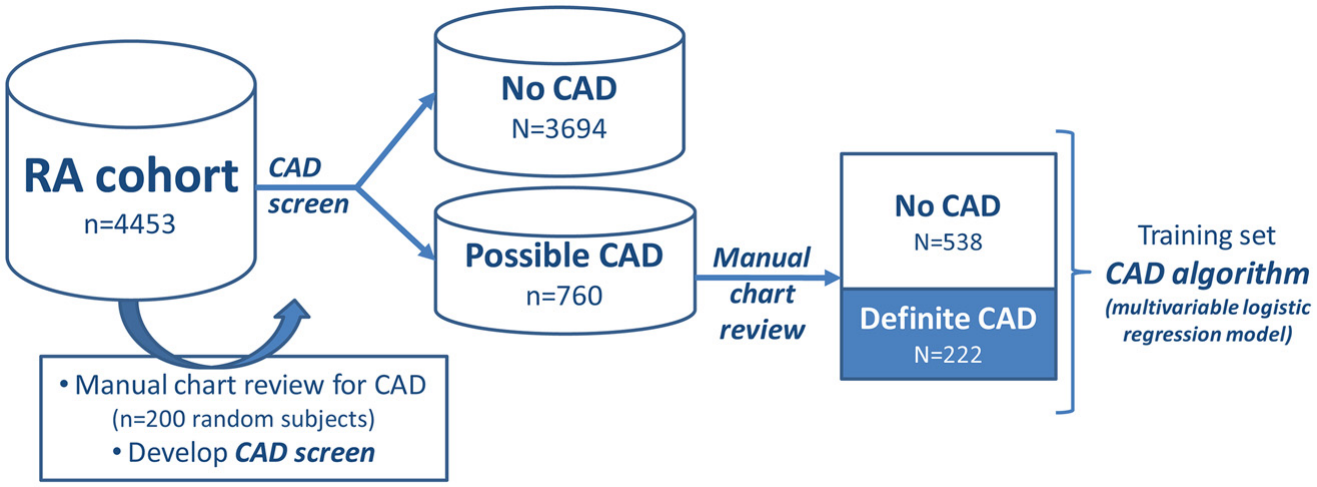
Overview of approach to developing the CAD algorithm in the RA cohort.

The RA cohort served as the training set for the CAD algorithm because of the low predicted CAD prevalence in this population. Generally, it is more challenging to develop robust algorithms with a high PPV (high accuracy) when the prevalence of the phenotype is low compared to high. We randomly selected 200 subjects from the RA cohort for review of CAD outcomes and estimated a CAD prevalence of 5.0%. We developed a CAD screen separating the RA cohort into two groups: the first group includes subjects with any possibility of CAD (possible CAD), and the second includes subjects with no evidence of CAD on chart review (Fig 1). The goal of the CAD screen was to maximize the negative predictive value (NPV) with a goal NPV of ≥99%. The CAD screen included≥1 ICD9 code for CAD (410.x, 411.x, 412.x, 414.x, 413.x) or ≥1 NLP mention for any CAD related concepts: CAD, CAD procedures, CAD biomarkers, positive stress test (S1 Appendix). This CAD screen achieved an NPV of 100% in the RA cohort.

We created a gold standard for CAD by reviewing the medical records of all RA subjects with ‘possible CAD’ based on the CAD screen (n = 760). The variables considered for the CAD algorithm are listed in S1 Appendix. The structured and NLP variables predictive for CAD were determined using logistic regression with adaptive LASSO using the BIC as the criterion for selecting an optimal penalty parameter [14].

#### Validation- IBD and DM cohorts

**Fig 2:**
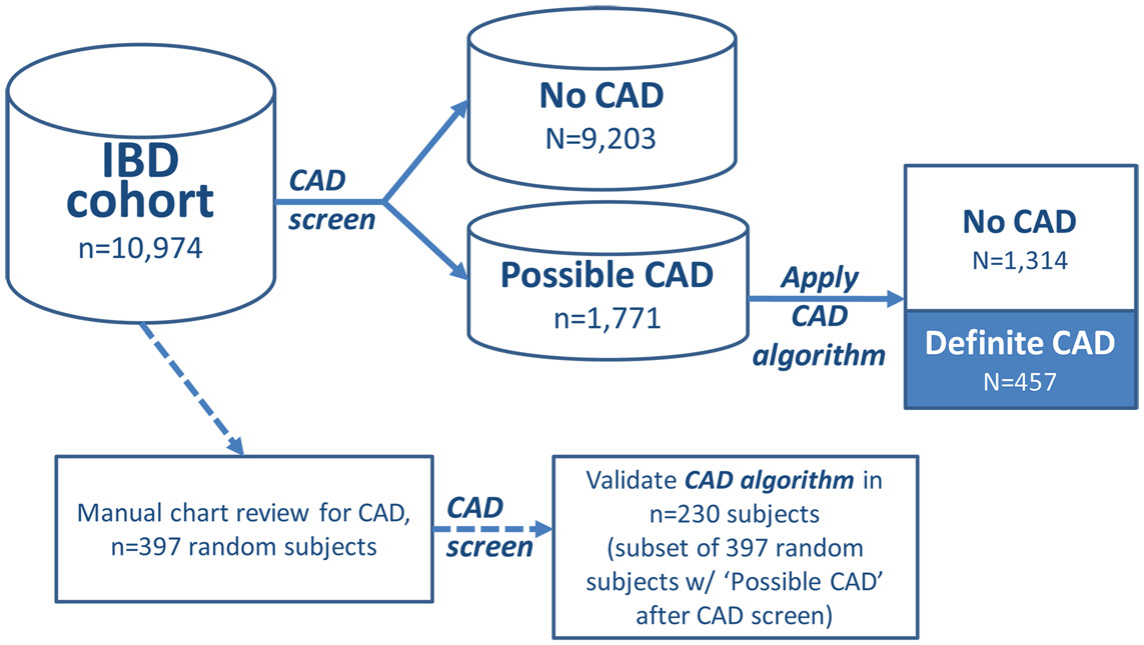
Validation of the CAD algorithm in the IBD cohort.

We validated the CAD screen and algorithm in the IBD and DM cohorts (Fig 2) by randomly selecting subjects from each cohort (n = 397, IBD and n = 362, DM) and reviewing charts for presence of CAD. On chart review, the prevalence of CAD in the IBD training set was 5.0% for IBD and 25.4% for DM. We applied the CAD screen on the IBD and DM datasets to calculate the NPV of the screen. The CAD screen was then applied to the total IBD (n = 10,974) and DM cohort (n = 65,099) to broadly group subjects into ‘possible’ and ‘no’ CAD. All subjects with ‘possible CAD’ were further evaluated using the CAD algorithm.

The CAD algorithm provided an estimate for the probability of CAD for each subject between 0 and 1.0. We classified subjects as CAD+ if their predicted probability is higher than a cut-off value *C*, where the cohort-specific *C* is the probability of having CAD ≥90% among those classified as CAD+ within each of the cohort.

Setting the PPV at 90% allows the likelihood of CAD to be equal across the 3 cohorts. Using 90% PPV as a point of comparison, we constructed, 2x2 contingency tables to calculate the sensitivity, specificity, and NPV for subjects classified with CAD from the algorithm compared to medical record review (using the random subset of patients whose medical records were reviewed, n = 230, IBD and n = 161, DM).

#### Performance of structured vs structured + NLP algorithm

We compared the performance characteristics of the CAD algorithm using structured only vs structured + NLP data for the IBD and DM cohorts. We calculated the sensitivity, specificity, NPV when the algorithm was targeted to a PPV of 90%. We also calculated the performance characteristics when ≥2 ICD9 codes for ischemic heart disease (S1 Appendix) were used to define CAD.

### Clinical example: risk of CAD in IBD and RA compared to DM

We conducted a preliminary cross-sectional association study comparing risk of CAD across DM, IBD and RA. To compare CAD across the cohorts, we used the same algorithm targeted at a PPV of 90% in each cohort. This ensures that the likelihood of CAD was common across the cohorts. For all subjects we extracted data on demographics: age, gender, and self-reported race. Using ICD9 codes, we assessed for the presence of CV risk factors including hypertension (HTN), diabetes mellitus (DM)[15, 16], and hyperlipidemia. Smoking status was obtained using NLP [17] (covariate details in S1 Appendix). We constructed a logistic regression model to determine the odds ratios for CAD across the cohorts, RA and IBD using DM as the reference. This model was adjusted by age, gender, race, HTN, hyperlipidemia and smoking status.

The Partners HealthCare Institutional Review Board approved all aspects of this study, including the waiver of individual written consent for use of de-identified EMR data for research.

## Results

### CAD algorithm

We developed a CAD EMR classification algorithm in an RA cohort (n = 4,453) and validated the algorithm in an IBD (n = 10,974) and DM cohort (n = 65,099). An overview of the basic demographic and CAD risk factors is shown in Table 1. The disease specific clinical characteristics have been previously published for RA and IBD. Briefly, in RA, 63% were ACPA positive, 60% had ≥1 electronic prescription for methotrexate and 33% for a tumor necrosis factor antagonist [8]. In IBD, 49.8% have Crohn’s Disease (CD), 49.9% have Ulcerative Colitis (UC), and 0.3% had both diseases [7]; approximately 21% of CD and 12% of UC patients had at least one bowel surgery. In DM, the mean hba1c was 7.6% and 33.8% of the patients had > 1 electronic prescription for insulin and 40% for an angiotensin converting enzyme inhibitor (Kumar, et al., in preparation).

**Table 1:** Clinical characteristics of subjects in the RA, IBD and DM cohorts.

Clinical characteristics	RA, n = 4453	IBD, n = 10,974	DM, n = 65,099
Age, mean (SD)	60.9 (14.8)	47.3 (18.8)	64.6 (15.4)
Female gender, %	79.1	53.2	46.9
Race, n (%)			
White	66.9	85.3	67.2
Black	5.7	3	12.1
Hypertension, n (%)	38.4	25.7	80.6
Diabetes mellitus, n (%)	14.4	9.2	92.1
Hyperlipidemia, n (%)	29.1	23.1	68
Ever smoker, n (%)	48.5	59	73.2
Mean f/u time in EMR, yrs (SD)	8.6 (5.5)	7.1 (5.7)	8.1 (5.9)

We observed that the most predictive variables to classify CAD in our EMR based cohorts was an NLP mention of coronary artery disease, total number of ICD9 codes, and ICD9 codes for ischemic heart disease (Table 2, S1 Appendix). The CAD screen developed in the RA cohort (NPV 100%) achieved an NPV of 100% when applied to the IBD cohort and 99% in the DM cohort. The CAD algorithm performed well with a specificity of 99.6% in IBD and 96.3% in DM (Table 3). An algorithm using only structured data had a similar specificity as an algorithm utilizing both structured and NLP data. The advantage of adding NLP derived variables was illustrated by the gains in the sensitivity of the algorithm: 14% in IBD and 3% in DM with the same accuracy (90% PPV). In IBD, the improvement in sensitivity resulted in classification of 67 more CAD cases (from 390 to 457), a 17.2% increase and in DM, 1,570 more CAD cases (15,392 to 16,962), a 10.2% increase. In comparison, simply applying ≥2 ICD9 codes for ischemic heart disease achieved PPVs of 40.9% in IBD, 47.3% in RA and 65.4% in DM.

**Table 2:** Variables in the final CAD algorithm.

Variable*	Variable type		Standardized coefficient	Standard error
	Structured	NLP		
Coronary artery disease		✓	1.44	0.19
ICD9 codes, normalized	✓		0.4	0.35
Ischemic heart disease	✓		0.35	0.17
CAD procedures		✓	0.33	0.16
EMR follow-up time (months)	✓		0.22	0.12
Coronary artery disease	✓		0.22	0.15
CABG, PCI	✓		0.20	0.24
No LDL values in EMR	✓		0.10	0.11
Age	✓		0.10	0.10
Mean LDL	✓		-0.01	0.08
Never smoker		✓	-0.01	0.10
Current smoker		✓	-0.07	0.11
Echocardiogram performed	✓		-0.16	0.13
Hypertension	✓		-0.19	0.16
ICD9 codes, total number	✓		-0.63	0.23
Intercept			-10.19	4.61

*Please refer to S1 Appendix for full description of variable

**Table 3:** Validation of accuracy of the two step classification of CAD (screening + algorithm) using structured data only, compared with the structured data + NLP. Natural language processing = NLP; negative predictive value = NPV; positive predictive value = PPV.

Disease cohort	Sensitivity	Specificity	PPV	NPV	Additional subjects classified with CAD (%)
IBD, structured data only	59	99.6	90	98.1	ref
IBD, structured + NLP	73	99.6	90	98.6	17.2
DM, structured data only	84	96.2	90	93.8	ref
DM, structured + NLP	87	96.3	90	94.5	10.2

### Risk of CAD in IBD and RA compared to DM

Applying a common CAD algorithm, targeted at a 90% PPV ensured a similar likelihood of CAD across the 3 cohorts. Using this definition, we conducted a preliminary study of CAD across the DM, IBD and RA. The prevalence of CAD was lowest in IBD (4.2%), followed by RA (5.0%) and was highest in DM (26.1%). The clinical characteristics of subjects stratified by CAD status is shown in Table 4. Across cohorts, subjects with prevalent CAD had a similar ages ranging from 71.3 to 72.9 years.

**Table 4:** Clinical characteristics of subjects classified with CAD in the RA, IBD and DM cohorts (PPV of CAD classification > = 90% PPV*).

Clinical characteristics	RA, n = 4453		IBD, n = 10,974		DM, n = 65,099	
	CAD yes, n = 245 (5.0%)	CAD no, n = 4208	CAD yes, n = 457 (4.2%)	CAD no, n = 10,517	CAD yes, n = 16,962 (26.1%)	CAD no, n = 48,136
Mean age, (SD)	72.9 (10.1)	60.2 (14.7)	71.3 (11.0)	46.3 (18.4)	71.7 (11.1)	62.1 (15.9)
Male gender (%)	45.7	19.4	70.7	45.7	66.9	48.3
Race (%)						
White	80.0	66.1	93.0	85.0	78.7	63.7
Black	7.4	5.6	2.2	3.0	7.1	14.0
Comorbidity						
Hypertension (%)	88.2	35.5	85.6	23.1	92.2	75.4
Diabetes mellitus (%)	40.0	12.2	35.2	7.1	N/A	N/A
Hyperlipidemia	82.5	26.0	81.0	20.6	83.6	61.5
Smoking status, ever vs never (%)	66.9	30.1	75.7	33.2	68.8	45.9
Follow-up (months), mean (SD)	139.7 (58.5)	101.2 (65.5)	112.9 (75.0)	83.7 (68.1)	98.1 (69.6)	96.7 (71.0)

*Selected specificity cutoff for each cohort based on PPV> = 90%; RA based on medical record review.

IBD and RA patients had a lower risk of CAD compared to DM. The difference in CAD risk between IBD and RA was lower compared to DM; the risk of CAD was 66% lower in IBD compared to DM, and 63% lower in RA compared to DM in the fully adjusted model (Table 5). Across all 3 cohorts, the traditional cardiovascular risk factors, hyperlipidemia, HTN, and ever smokers, were significantly associated with higher risk of CAD. Compared to RA, IBD subjects has a trend for lower risk of CAD with a 16% lower risk for CAD compared to RA (p = 0.06).

**Table 5:** Unadjusted and adjusted odds ratios comparing risk of CAD in IBD and RA to DM.

Clinical variables	Unadjusted OR (95% CI)	Adjusted OR (95% CI)
Age	-	1.05 (1.05, 1.05)
Male gender	-	2.27 (2.18, 2.36)
Hyperlipidemia	-	2.48 (2.36, 2.61)
Ever smoker	-	1.92 (1.84, 1.99)
Hypertension	-	1.80 (1.68, 1.91)
**IBD vs DM**	0.12 (0.11, 0.14)	**0.34 (0.31, 0.37)**
**RA vs DM**	0.17 (0.15, 0.19)	**0.37 (0.32, 0.43)**

## Discussion

Existing EMR phenotype algorithms are typically designed for use in populations similar to the derivation cohort. In this study, we developed a CAD algorithm designed for portability across diverse populations to allow for comparison of risk and risk factors across diseases. A major difference between the DM, IBD and RA cohorts was the prevalence of CAD. Since low prevalence can limit the accuracy of an algorithm, successfully phenotyping CAD across these populations required the use of a sensitive CAD screen which included NLP (with high NPV) to initially separate patients into ‘possible CAD’ and those with ‘no CAD’ data in the EMR. The CAD algorithm was then applied to all subjects with ‘possible CAD’, providing a probability of CAD for each patient.

Including NLP into the CAD algorithm improved the sensitivity of the algorithm in both IBD and DM, with the greatest gains in IBD (14% increase in subjects classified with CAD at 90% PPV). In contrast, in DM where the prevalence of CAD was higher, the improvements in sensitivity with the addition of NLP were lower (increase of 3%). These data corroborate with findings from previous EMR phenotype algorithm studies where we observed that NLP can simultaneously improve the accuracy and sensitivity of the phenotype algorithm [7, 8]. We believe this occurs not because the NLP data are necessarily more accurate than the structured data, rather that the additional data extracted using NLP adds to or enriches information captured using structured data. As an example, both the structured data for CABG and NLP data for CABG were informative for classifying CAD.

In our clinical example, we compared CAD risk in IBD and RA to DM. We note that in a typical application of a phenotype algorithm, investigators maximize the PPV of the algorithm for their particular cohort. In this example, the PPV must be the same across all 3 cohorts. In our example, we selected a PPV of 90%. This ensures that the likelihood of having CAD, the outcome of interest, is the same across the 3 cohorts which enabled us to compare CAD risk across the population. The ability to tune the PPV is an important feature of the algorithm, and setting one PPV is a key aspect of the study design.

The preliminary analysis also touches upon a scientific debate regarding whether inflammatory diseases should be considered CAD risk equivalents [18]. Several studies have compared the risk of CAD in RA with DM. Two published studies suggest that RA patients are at equivalent or higher risk of CAD than patients with diabetes [10, 11]. One recent study found that CAD risk is lower in RA compared to DM [19]. Our findings are consistent with the more recent studies, where we observed that patients with diabetes were at highest risk for CAD, followed by RA, and IBD had the lowest risk for CAD. The relative differences in CAD risk are in line with population based studies that compare CAD risk of each disease with the general population. Patients with diabetes are at 2 to 4 fold or higher increased risk of CAD than the general population [20, 21]. In RA, multiple studies have demonstrated that the risk of CAD is estimated to be 1.5 to 2 fold increased risk compared to the general population [22, 23]. Finally, in IBD, a recent meta-analysis observed found a 1.2 fold increased risk for MI compared to non-IBD [24]. Thus, although CAD risk is a major cause of morbidity and mortality in IBD and RA patients, the magnitude of risk does not appear to be equivalent to patients with DM. Future studies include a more detailed investigation of these findings.

There are limitations to this study. This study may not be generalizeable to community based patient populations as the study is based in the EMR shared by two large tertiary care centers. Ascertainment and misclassification bias was a concern. In our analyses we assumed that the absence of a CV risk factor or CAD diagnosis was the absence of disease when in fact the diagnos(es) may have been made outside our healthcare system and therefore not captured in the EMR. Differences in how CV risk factors are recorded across cohorts could lead to ascertainment bias. Despite this potential pitfall, we found a consistent relationship between relative risk across DM, IBD and RA with population based studies studying each specific disease and risk of CAD compared to the general population. The association between CV risk factors and CAD was also consistent with prior literature, e.g. hyperlipidemia is associated with a higher risk of CAD. Our algorithms achieved a PPV of 90% which can lead to misclassification bias in an estimated 10% of patients. The effect of misclassification in the clinical example would likely bias our findings towards finding no differences between the cohorts. We performed a preliminary cross-sectional study the association between 3 chronic diseases and CAD risk. Thus, a diagnosis of DM, IBD or RA may have occurred after CAD. In general, traditional cardiovascular risk factors, particularly DM generally precede diagnosis of CAD [21]. The peak age of onset for IBD is 15–29 years [25] which in most cases precedes development of CAD. While RA can occur at any age, the increased risk for CAD appears to occur after diagnosis of RA [26].

In conclusion, we demonstrate the methods for development, validation and implementation of an EMR based CAD classification algorithm in diverse patient populations with varying CAD prevalence. NLP was particularly important in accurately classifying additional subjects with CAD in cohorts where the prevalence of CAD was low, e.g. IBD. After applying a common EMR CAD algorithm, we provide preliminary data showing that the risk of CAD was 63–68% lower in RA and IBD compared to DM. More studies are needed to further define the differences in CAD risk across diverse patient populations, and investigate the components of inflammation that may be contributing to these variations in risk.

## Supporting Information

S1 Appendix (DOCX)
