# Supplementary material for: Methods to Develop an Electronic Medical Record Phenotype Algorithm to Compare the Risk of Coronary Artery Disease across 3 Chronic Disease Cohorts
Source: PLoS One. 2015 Aug 24;10(8):e0136651. doi: 10.1371/journal.pone.0136651 (PMC4547801; doi:10.1371/journal.pone.0136651)
Supplement: S1 Appendix — (DOCX) [file pone.0136651.s001.docx]

**Appendix**

**Appendix 1.** Detailed definitions for (A) structured and (B) NLP concepts used in the study.

| **(A) Name of structured variable** | **Description** |
| --- | --- |
| Beta-blocker | EMR prescription -> Cardiovascular agents -> beta-adrenergic blocking agents -> cardioselective beta blockers |
| Coronary artery bypass graft (CABG) or percutaneous coronary intervention (PCI) | CABG  CPT: 33510, 33518, 33519, 33533, 33534, 33536, 33572, 35500  ICD9: 36.10, 36.11, 36.12, 36.13, 36.14, 36.15, 36.16, 36.17, 36.19)  PCI  CPT: 92980, 92981, 92982, 92984, 92995, 92996, 92998  ICD9: 0.66, 36.01, 36.02, 36.05, 36.06, 36.07, 36.09 |
| Coronary artery disease (CAD) | ICD9 410.x, 411.x, 412.x, 414.x, 413.x |
| Diabetes mellitus (DM) | ICD9 250.xx |
| Dyslipidemia | ICD9 272.0-272.4 |
| Echocardiograms | Number of echocardiograms performed |
| EMR follow-up time (months) | Number of months between date of first record in EMR to date of last record in EMR |
| Hypertension (HTN) | ICD9 401.x, 402.x. 403.x. 404.x, 405.x |
| ICD9 codes | Number of total ICD9 codes; ICD9 codes normalized is the natural log of the ICD9 total codes |
| Ischemic heart disease (IHD)* | ICD9 410.x, 411.x, 412.x, 414.x |
| Low density lipoprotein (LDL) | Presence or absence of LDL values in EMR; mean values calculated from all available LDL values |

*Difference between CAD and IHD is that CAD includes angina pectoris (413.x)

| **(B) NLP concept extracted from narrative data** | **Examples of terms describing concept** |
| --- | --- |
| Coronary artery disease | Ischemic heart disease  Myocardial infarction  Ischemic cardiomyopathy  Heart attack  Coronary artery disease  CAD, s/p stent  Coronary heart disease  Coronary blockage  Non ST elevation myocardial infarction  ST elevation myocardial infarction  Coronary stenosis  Cardiac arrest  Sudden cardiac death |
| CAD procedures | Coronary artery bypass graft  Left heart catheterization  Percutaneous coronary intervention  Percutaneous angioplasty  Cardiac catherization with:  Stent  Balloon angioplasty  Angioplasty  Plain old balloon angioplasty |
| CAD biomarkers | Elevated or positive troponin, cardiac biomarkers, cardiac enzymes, MB, CKMB, CK-MB |
| Positive stress test | Perfusion defect, perfusion abnormality  Reversible defect  Irreversible defect  Fixed defect  Positive stress test, MIBI, SPECT, ETT-MIBI, EKG-MIBI |
| Ever smoker | Smoking module classifies subjects as current, past and never smoker  Ever smoker= current or past smoker  Never smoker= never smoker |
